# The efficacy and feasibility of total reconstruction versus nontotal reconstruction of the pelvic floor on short-term and long-term urinary continence rates after radical prostatectomy: a meta-analysis

**DOI:** 10.1186/s12957-017-1296-z

**Published:** 2017-12-20

**Authors:** Yu-Peng Wu, Ning Xu, Shi-Tao Wang, Shao-Hao Chen, Yun-Zhi Lin, Xiao-Dong Li, Qing-Shui Zheng, Yong Wei, Xue-Yi Xue

**Affiliations:** Department of Urology, The First Affiliated Hospital of Fujian Medical University, 20 Chazhong Road, Fuzhou, 350005 China

**Keywords:** Total reconstruction, Pelvic floor, Urinary continence, Radical prostatectomy

## Abstract

**Background:**

Recently, total pelvic floor reconstruction (TR) has been the treatment of choice for improving urinary incontinence (UI) after radical prostatectomy (RP). However, the superiority of TR with respect to urinary continence recovery following RP remains controversial. This study identified the effect of TR versus nonTR of the pelvic floor on short-term and long-term continence rates after RP.

**Methods:**

A literature search was performed in November 2017 using the PubMed, Embase, and Web of Science databases. Only comparative research or clinical studies reporting urinary continence outcomes was included in the meta-analysis, and the quality of evidence was evaluated using the 2011 Level of Evidence for therapy research.

**Results:**

We analyzed ten studies reporting urinary continence rates after RP at one or more postoperative time points (1, 2, 4, 12, 24, and 52 weeks). TR was associated with significantly better urinary continence outcomes at 1 week (OR 2.76, 95% CI 1.58–4.84, *P* < 0.001), 2 weeks (OR 2.57, 95% CI 1.74–3.80, *P* < 0.001), 4 weeks (OR 2.61, 95% CI 1.56–4.38, *P* < 0.001), 12 weeks (OR 4.33, 95% CI 2.01–9.33, *P* < 0.001), 24 weeks (OR 3.83, 95% CI 1.54–9.55, *P* = 0.004), 52 weeks (OR 4.10, 95% CI 1.80–9.38, *P* < 0.001) after RP. There was no difference in the rate of complications between the two arms (OR 0.54, 95% CI 0.19–1.54, *P* = 0.25).

**Conclusions:**

Compared with nonTR, TR is significantly and positively associated with a return to continence but not with complication rate in men following RP, suggesting that TR may be useful for decreasing the urinary incontinence rate after surgery.

## Background

Radical prostatectomy (RP) remains the standard surgical strategy for localized prostate cancer [1, 2]. Urinary incontinence (UI) is one of the most distressing complications of RP. The long-term continence rate varies from 66.7 to 97% at 48 weeks after laparoscopic radical prostatectomy (LRP) and robot-assisted radical prostatectomy (RARP). However, the short-term continence rate varies from 17 to 89% at 12 weeks [3, 4]. Apparently, achieving short-term continence is challenging and has a great impact on the health-related quality of life (QoL) of patients [5, 6].

Recently, several technical modifications to improve postoperative continence have been described, including intraoperative maximization of membranous urethral length [7], the dorsal vein complex-preserving technique [8], intrafascial nerve-sparing [4, 9, 10], preservation of the puboprostatic collar [11], anterior reconstruction [12, 13], posterior reconstruction [14–16], and anterior plus posterior reconstruction [17, 18]. The anterior or posterior reconstruction and total pelvic floor reconstruction (TR) (anterior plus posterior reconstruction) techniques are reported to be simpler than the others among these modifications. The surgical techniques in different studies are varied (Table 1). In a study by Hoshi et al., the TR technique was described in two parts: anterior reconstruction of the detrusor apron and posterior reconstruction of the musculofascial plate [10]. TR has become widespread because this technique is simpler and has a favorable effect on early recovery from incontinence. The time to return to continence after RP in published studies varies [9, 18–20]. Four previous randomized controlled trials (RCTs) showed inconsistent results 4 weeks after surgery. Hurtes et al. [20] and Koliakos et al. [21] demonstrated TR had a statistically significant advantage. In contrast, Menon et al. [22] demonstrated similar outcomes in participants who received TR management and those who received nonTR management. Moreover, Hoshi et al. [10] demonstrated a significant advantage in favor of TR in terms of long-term (48 weeks) continence rates. However, Sammon et al. [23] showed TR did not result in improvement in long-term continence rates. Therefore, we performed a meta-analysis to evaluate the effect of TR versus nonTR management on short- and long-term continence rates after RP.

**Table 1:** Preserved relevant anatomy facilitating reconstruction

Study ID	Puboprostatic ligaments	Pubovesical collar	Arcus tendineus	Rhabdosphincter	Denonvillier’s fascia	Bladder neck	Median dorsal raphe
Student [27], 2017	+	–	+	+	+	+	+
Liao [19], 2016	+	–	+	+	+	+	+
Atug [25], 2012	–	–	–	+	+	+	–
Hoshi [10], 2014	+	–	–	+	+	+	+
Hurtes [20], 2012	+	–	–	+	+	–	+
Menon [22], 2008	+	+	–	+	+	–	+
Tewari [18], 2007	+	–	+	+	+	+	+
Koliakos [21], 2009	+	–	–	–	+	+	+
Tan [26], 2010	+	–	+	+	+	+	+
Sammon [23], 2010	+	+	–	+	+	–	–

## Methods

### Ethics statement and objective

Ethical approval was not required for this meta-analysis because it did not affect the participants directly. The aim of this meta-analysis was to identify the effect of TR versus nonTR management on short- and long-term urinary continence rates after RP.

### Search strategy

PubMed, Embase, and Web of Science databases were searched for relevant articles from the inception of each database through November 2, 2017. The PubMed was searched using the combined terms “prostatectomy OR radical prostatectomy AND urinary continence AND anterior or posterior OR total OR complete AND reconstruction OR restoration OR anastomosis OR fixation OR puboperineoplasty in the title and abstract. Embase and Web of Science databases were searched using the same combined terms, keywords, and search strategy.

### Study selection

Two investigators (Yu-Peng Wu and Shi-Tao Wang) extracted data employing a predefined data extraction form. Subsequent full-text record screening was performed independently by two investigators (Yu-Peng Wu and Shi-Tao Wang). Disagreements were resolved by a third reviewer (Ning Xu) [1]. Full-text articles were obtained to determine eligibility when the information from the title or abstract was insufficient. Reference lists of relevant studies were also manually searched to identify articles not found in the search strategies. Studies were included and excluded according to the criteria presented in Table 2.

**Table 2:** Inclusion and exclusion criteria

Inclusion criteria	Exclusion criteria
Men undergoing RP	Review articles and descriptive commentaries
Studies reporting TR versus nonTR	Animal studies
Postoperative continence assessment completed	Conference abstracts or poster publications
English language	Published in a language other than English
Full journal article publication in a peer-reviewed journal	

### Quality assessment

All included studies were categorized based on the 2011 Level of Evidence for therapy research as a systematic review of randomized trials (level 1); randomized trial or observational study with dramatic effects (level 2); nonrandomized controlled cohort/follow-up study (level 3); case series, case-control study, or historically controlled study (level 4); or mechanism-based reasoning (level 5) [24].

### Data extraction and synthesis

Data extracted from each comparative study included study characteristics and preoperative parameters, perioperative outcome measures, complications, and pathological results continence definition, data collection, and, when available, the 1-, 2-, 4-, 12-, 24, and 52-week urinary continence rates.

### Meta-analysis methods

Meta-analysis was conducted using the RevMan 5.3 software (Cochrane Collaboration, Oxford, UK). Statistical heterogeneity was evaluated using the chi-square test. If no heterogeneity existed when *P* > 0.1 and *I* ^2^ < 50%, a fixed-effects model was applied to pool the trial results. Significant heterogeneity was identified if *P* < 0.1 and *I* ^2^ > 50%, and a random-effects model was employed [2]. Sensitivity analysis was then done to determine whether the use of the excluded study would alter the results substantially. The cumulative outcomes of dichotomous variables were determined using odds ratios (ORs) and 95% confidence intervals (CIs). *P* < 0.05 was considered statistically significant.

## Results

### Literature search

**Fig. 1:**
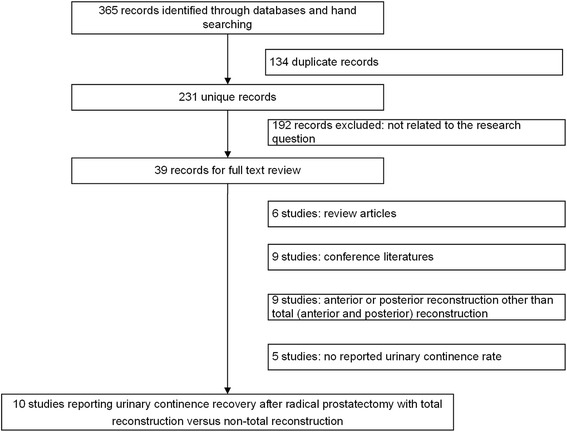
Flow chart demonstrating selection of studies for review

Figure 1 shows the PRISMA flow diagram for the study selection process. The searches retrieved 365 citations. After removal of duplicates and review of the abstracts and full-text articles, ten studies including 12 trials were eligible for inclusion in this meta-analysis. All corresponding authors were contacted via email to provide clarification and/or additional data when necessary. At least three follow-up attempts were made for queries sent; unfortunately, these attempts were unsuccessful.

### Quality assessment

The remaining 12 trials included 7 RCTs (58.3%) (level 2), 2 prospective comparative trials (PTs) (16.7%) (level 3), and 3 retrospective comparative trials (RTs) (25.0%) (level 4).

### Characteristics of the studies included

#### Patient and surgical characteristics

Participant and surgical characteristics are presented in Tables 3, 4, and 5. A total of ten studies [10, 18–23, 25–27] comprising 12 trials reported the continence rate. For the study reporting the continence rate at 1 week, a total of five studies [18, 19, 22, 25, 26] comprising six trials were included; urinary continence was defined as 0 pads in two trials [19, 22] and as 0 pads or 1 pad used for safety (0–1 for safety) in four trials [18, 22, 25, 26]. For the study reporting the continence rate at 2 weeks, a total of four trials [19, 20, 25, 27] were included; urinary continence was defined as 0 pads in three trials [19, 20, 27] and 0–1 for safety in one trial [25]. For the study reporting the continence rate at 4 weeks, a total of ten studies [10, 18–23, 25–27] comprising 12 trials were included; urinary continence was defined as 0 pads in five trials [19, 20, 22, 23, 27] and 0–1 for safety in seven trials [10, 18, 21–23, 25, 26]. For the study reporting the continence rate at 12 weeks, a total of six trials [10, 18–20, 25, 26] were included; urinary continence was defined as 0 pads in two trials [19, 20] and 0–1 for safety in four trials [10, 18, 25, 26]. For the study reporting the continence rate at 24 weeks, a total of seven trials [10, 18–20, 25–27] were included; urinary continence was defined as 0 pads in three trials [19, 20, 27] and 0–1 for safety in four trials [10, 18, 25, 26]. For the study reporting the continence rate at 52 weeks, a total of five trials [10, 18, 19, 25, 26] were included; urinary continence was defined as 0 pads in one trial [19] and 0–1 for safety in four trials [10, 18, 25, 26].

**Table 3:** Study characteristics and preoperative parameters

Study ID	Country	Method	Sample		Age^*^, year	Prostate volume^*^, mL	IIEF-5 score, median (IQR)	IPSS	BMI^*^ (kg/m^2^)	Preoperative PSA^*^, ng/mL	Nerve-sparing procedure, *n* (%)		
											Bilateral	Unilateral	Non
Student [27], 2017	Czech Republic	RARP	T	32	64.5 (56.0–67.0)	35.0 (30.0–55.0)	19.0 (13.5–21.0)	5.5 (2.0–9.3)	27.7 (25.7–33.2)	7.3 (5.2–11.2)	20 (62.5)	6 (18.75)	6 (18.75)
			N	34	62.5 (61.0–68.0)	32.5 (22.0–52.0)	17.0 (13.3–21.0)	5.0 (2.8–14.0)	28.0 (25.4–31.4)	5.7 (3.6–12.0)	23 (67.6)	5 (14.7)	6 (17.6)
Liao [19], 2016	China	LRP	T	82	65.79 ± 7.27	42.06 ± 19.34^*^	–	7 (0~18)	24.26 ± 2.88	33.07 ± 40.65	27 (32.92)	19 (23.17)	36 (43.90)
			N	79	67.88 ± 6.65^*^	41.92 ± 14.76^*^	–	6 (0~19)	25.35 ± 2.13	26.86 ± 31.95	16 (20.25)	28 (35.4)	35 (44.3)
Atug [25], 2012	Turkey	RARP	T	125	61.5 (9.5)	50 (24)	–	–	27 (4)	5.62 (3.65)	89 (71.2)	21 (16.8)	15 (12)
			N	120	60.50 (11)	49.5 (25)	–	–	28 (4)	5.49 (2.92)	93 (77.5)	22 (18.3)	5 (4.2)
Hoshi [10], 2014	Japan	LRP	T	81	65.2 ± 5.6	–	–	–	–	9.2 ± 5.7	–	–	–
			N	47	65.4 ± 4.4	–	–	–	–	11.8 ± 9.5	–	–	–
Hurtes [20], 2012	France	RARP	T	39	62.5 ± 6.8	38.5 (20–90)	20 (6–25)	5 (0–16)	26.3 ± 3.5	6.4 (2.7–16.6)	–	–	–
			N	33	62.4 ± 5	40 (25–80)	23 (1–25)	7 (0–17)	25.7 ± 3.6	7.9 (3.6–23.6)	–	–	–
											Standard^^^	Veil^#^	
Menon [22], 2008	America	RARP	T	59	–	45.2^*^	–	–	27.9	6.1	37 (63)	22 (37)	–
			N	57	–	63.4^*^	–	–	27.9	6.4	31 (54)	26 (46)	–
Tewari [18], 2007	America	RARP	T	182	61.21	50.53	–	–	24.98	5.76	–	–	–
			N	214	64.32	57.12	–	–	28.77	6.02	–	–	–
Koliakos [21], 2009	Belgium	RARP	T	23	60.96 ± 6.56	–	–	–	–	10.43 ± 3.37	–	–	–
			N	24	61.75 ± 5.96	–	–	–	–	10.47 ± 2.22	–	–	–
Tan [26], 2010	America	RARP	T	1383	60.3 ± 7.4	46 (38–56)	–	7 (3–12)	26.77 ± 3.59	4.8 (3.7–6.57)	–	–	–
			N	214	59.8 ± 6.6	48 (39–63)	–	6 (2–11.25)	28.34 ± 5.57	4.8 (3.9–6.7)	–	–	–
											Standard^^^	Veil^#^	–
Sammon [23], 2010	America	RARP	T	46	59.9 ± 7.4	–	–	–	28.3 ± 4.1	5.8 ± 66.3	23 (21)	21 (45.7)	–
			N	50	60 ± 7.2	–	–	–	28 ± 4.21	5.7 ± 3.2	27 (54.0)	23 (46.0)	–

*SD* standard deviation, *IQR* interquartile range, *IIEF* International Index of Erectile Function 5, *IPSS* International Prostate Symptoms Score, *BMI* body mass index, *PSA* prostate specific antigen, *T* total reconstruction, *N* nontotal reconstruction, *LRP* laparoscopic radical prostatectomy, *RARP* robot-assisted radical prostatectomy

^*^Mean ± SD or median (IQR)

^^^Interfacial dissection: prostatic fascia excised with specimen

^#^Intrafacial dissection: prostatic fascia dissected off the prostate

**Table 4:** Perioperative outcome measures, complications, and pathological results

Study ID	Operative time^*^, min	Estimated blood loss^*^, mL	Duration of catheterization^*^, d	Complications, *n* (%)	PSM rate (%)		
					Total	pT2	pT3
Student [27], 2017	78.0 (63.0–126.0)^#^	145.0 (85.5–234.5)	–	2 (6.2)	4 (12.5)	–	–
	76.5 (48.0–130.0)^#^	140.0 (80.0–294.0)	–	2 (5.8)	5 (14.7)	–	–
Liao [19], 2016	147.33 ± 29.89	232.63 ± 217.93	13.96 ± 2.20	36 (43.90)	13 (15.85)	–	–
	130.81 + 21.66	225.42 ± 164.96	16.13 ± 16.47	35 (44.3)	10 (12.65)	–	–
Atug [25], 2012	175(55)	300 (200)	7 (0)	–	115 (92)	–	–
	200(70)	350 (250)	7 (1)	–	110 (91.7)	–	–
Hoshi [10], 2014	240 (139–575)	228 (59–1340)	–	3 (3.7)	13 (16.0)	8 (11.8)	5 (38.5)
	219(160–415)	236 (55–1875)	–	4 (8.5)	8 (17.0)	3 (7.7)	5 (62.5)
Hurtes [20], 2012	200 (100–400)	300 (100–1100)	6 (5–40)	8 (20.5)	8 (21.1)	–	–
	220 (105–350)	300 (20–1500)	7 (5–20)	6 (18.2)	4 (12.1)	–	–
Menon [22], 2008	171	–	7	–	–	–	–
	158	–	7	–	–	–	–
Tewari [18], 2007	104	140	7	–	–	7 (4.8)	–
	132	150	<7	–	–	7 (3.8)	–
Koliakos [21], 2009	126.52 ± 11.48	225.65 ± 80.95	6.26 ± 0.96	–	–	–	–
	124.54 ± 10.78	224.17 ± 64.06	6.17 ± 1.88	–	–	–	–
Tan [26], 2010	180 (150–230)	140 (140–150)	–	11 (0.8%)	–	65 (5.7)	–
	187 (160–240)	150 (150–162.5)	–	13 (6.0%)	–	6 (3.2)	–
Sammon [23], 2010	174.1 ± 38.4	–	7.7 ± 2.4	–	–	–	–
	165.2 ± 36	–	9.2 ± 4.2	–	–	–	–

*PSM* positive surgical margin

*Mean ± SD or median (IQR)

^#^Console time

**Table 5:** Clinical and pathological data of patients

Study ID		Clinical stage			Pathological stage				Gleason biopsy, *n* (%)			Gleason score, *n* (%)		
		cT1	cT2	cT3	pT0	pT1	pT2	pT3	< 7	7	> 7	< 7	7	> 7
Student [27], 2017	T 32	–	–	–	–	–	20 (62.5)	12 (37.5)	–	–	–	–	–	–
	N 34	–	–	–	–	–	23 (67.6)	11 (32.4)	–	–	–	–	–	–
Liao [19], 2016	T 82	–	–	–	3 (3.7)	53 (64.6)	26 (31.7)	0	–	–	–	38 (46.3)	32 (39.0)	12 (14.6)
	N 79	–	–	–	2 (2.5)	52 (65.8)	22 (27.8)	3 (3.8)	–	–	–	27 (34.2)	35 (44.3)	17 (21.5)
Atug [25], 2012	T 125	–	–	–	–	104 (82.9)	19 (15.5)	2 (1.6)	–	–	–	–	–	–
	N 120	–	–	–	–	100 (83.1)	17 (14.4)	3 (2.5)	–	–	–	–	–	–
Hoshi [10], 2014	T 81	36 (44.4)	40 (49.4)	5 (6.2)	–	–	68 (84.0)	13 (16.0)	29 (35.8)	36 (44.4)	16 (19.8)	34 (42.0)	35 (43.2)	12 (14.8)
	N 47	30 (63.8)	15 (31.9)	2 (4.3)	–	–	39 (83.0)	8 (17.0)	20 (42.6)	21 (44.7)	6 (12.8)	15 (31.9)	26 (55.3)	6 (12.8)
Hurtes [20], 2012	T 39	–	–	–	2 (5.1)	29 (74.4)	8 (20.5)	–	24 (61.5)	11 (28.2)	4 (10.3)	11 (28.2)	26 (66.7)	2 (5.1)
	N 33	–	–	–	0	24 (72.7)	9 (27.3)	–	18 (54.6)	11 (33.3)	4 (12.1)	12 (36.4)	18 (54.5)	3 (9.1)
Tewari [18], 2007	T 182	147 (80.8)	30 (16.5)	5 (2.7)	–	146 (80.28)	18.77	0.46	–	–	–	–	–	–
	N 214	161 (75.2)	51 (23.8)	2 (0.9)	–	186 (87.38)	11.21	0.93	–	–	–	–	–	–
Tan [26], 2010	T 1383	1250 (90.4)	133 (9.6)	0	–	1147 (82.9)	236 (17.1)	–	828 (59.9)	459 (33.2)	96 (6.9)	430 (31.1)	874 (63.2)	79 (5.7)
	N 214	161 (75.2)	51 (23.8)	2 (0.9)	–	186 (86.9)	28 (13.1)	–	156 (72.9)	48 (22.4)	10 (4.7)	102 (47.4)	100 (46.9)	12 (5.6)

### Definition of TR

The surgical technique for TR includes two components: one is a posterior reconstruction of the musculofascial plate and the other is an anterior reconstruction of the detrusor apron. In posterior reconstruction, the bladder neck, Denonvillier’s fascia, and the median dorsal raphe are sutured together before anastomosis. In anterior reconstruction, the bilateral puboprostatic ligaments and pudendal arteries are preserved, and the detrusor apron and puboprostatic ligament collar are reconstructed after vesicourethral anastomosis [10].

### Definition of UI

All studies reported the definition of continence and method of assessment used. Eleven of the 12 trials reported similar methods of assessing postoperative UI using questionnaires. Only one trial assessed the postoperative UI using interview data. Six studies [10, 18, 21, 23, 25, 26] comprising seven trials defined continence as 0–1 pad used, and five trials [19, 20, 22, 23, 27] defined continence as 0 pads used.

### Outcomes

Table 6 summarizes the data of the 12 trials comparing TR versus nonTR in terms of continence definition, method of data collection, and continence rates.

**Table 6:** Continence definition, data collection, and continence rates

Study ID	Study design	Continence definition	Data collection		Cases, *n*	1 week	2 weeks	4 weeks	12 weeks	24 weeks	52 weeks
Student [27], 2017	RCT	0 pad	Validated questionnaire	T	32	–	43.8	62.5	–	75.0	–
				N	34	–	11.8	14.7	–	44.1	–
Liao [19], 2016	Retrospective	No leak, total control, no pad	Validated questionnaire	T	82	13.41	32.92	65.85	81.71	90.24	95.12
				N	79	7.59	20.25	37.97	58.22	81.01	89.87
Atug [25], 2012	Retrospective	0–1 safety pad	Interview	T	125	71.2	72.8	80.8	84.8	86.4	91.2
				N	120	23.33	49.1	76.6	80.8	85.83	88.33
Hoshi [10], 2014	Retrospective	0–1 safety pad	Validated questionnaire	T	81	–	–	18.4	45.7	71.4	–
				N	47	–	–	4.5	26.1	46.8	–
Hurtes [20], 2012	RCT	No leak, total control, no pad	Validated questionnaire	T	39	–	5.9	26.5	45.2	65.4	–
				N	33	–	3.6	7.1	15.4	57.9	–
Menon1 [22], 2008	RCT	0–1 safety pad	Validated questionnaire	T	59	54	–	80	–	–	–
				N	57	51	–	74	–	–	–
Menon2 [22], 2008	RCT	0 pad	Validated questionnaire	T	59	20	–	42	–	–	–
				N	57	16	–	47	–	–	–
Tewari [18], 2007	Prospective	0–1 safety pad	Validated questionnaire	T	182	38.37	–	82.56	91.3	97.14	97.14^a^
				N	214	13.15	–	35.21	50.23	61.97	82.16
Koliakos [21], 2009	RCT	0–1 safety pad	Validated questionnaire	T	23	–	–	65	–	–	–
				N	24	–	–	33	–	–	–
Tan [26], 2010	Prospective	0–1 safety pad	Validated questionnaire	T	1383	30.8	–	70	91.7	95	98
				N	214	13.1	–	35.2	50.2	61.9	82.1
Sammon1 [23], 2010	RCT	0–1 safety pad	Validated questionnaire	T	46	–	–	42	–	–	–
				N	50	–	–	47	–	–	–
Sammon2 [23], 2010	RCT	0 pad	Validated questionnaire	T	46	–	–	80	–	–	–
				N	50	–	–	74	–	–	–

*RCT* randomized controlled trial, *weeks* week; *T* total reconstruction, *N* nontotal reconstruction

^a^The 52-week urinary continence rate was not reported directly in the study of Tewari, [18]. Thus, the urinary continence rate at 24 weeks was represented as the urinary continence rate at 52 weeks

#### Return of continence at 1 week

**Fig. 2:**
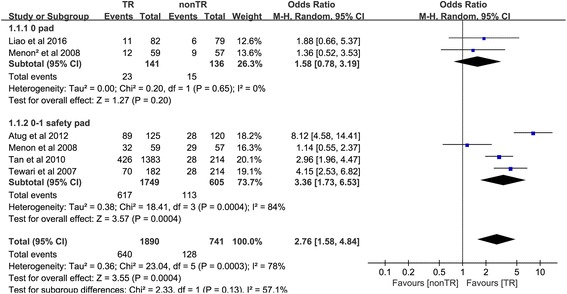
Forest plot of the odds of included studies comparing TR versus nonTR with respect to return to continence at 1 week. *CI* confidence interval, *OR* odds ratio, *RCT* randomized controlled trial, *TR* total pelvic floor reconstruction

Five studies [18, 19, 22, 25, 26] comprising six trials reported the number of people returning to continence at 1 week. In the 0-pad subgroup, the cumulative results showed no statistically significant difference between the TR and nonTR groups in terms of return to continence at 1 week (OR 1.58, 95% CI 0.78 to 3.19; *P* = 0.20). In the 0–1 for safety pad subgroup, the cumulative results showed a statistically significant difference in favor of TR 1 week after RP (OR 3.36, 95% CI 1.73 to 6.53; *P* < 0.001). The overall cumulative results showed a statistically significant difference in favor of TR 1 week after RP (OR 2.76, 95% CI 1.58–4.84; *P* < 0.001) (Fig. 2).

#### Return to continence at 2 weeks

**Fig. 3:**
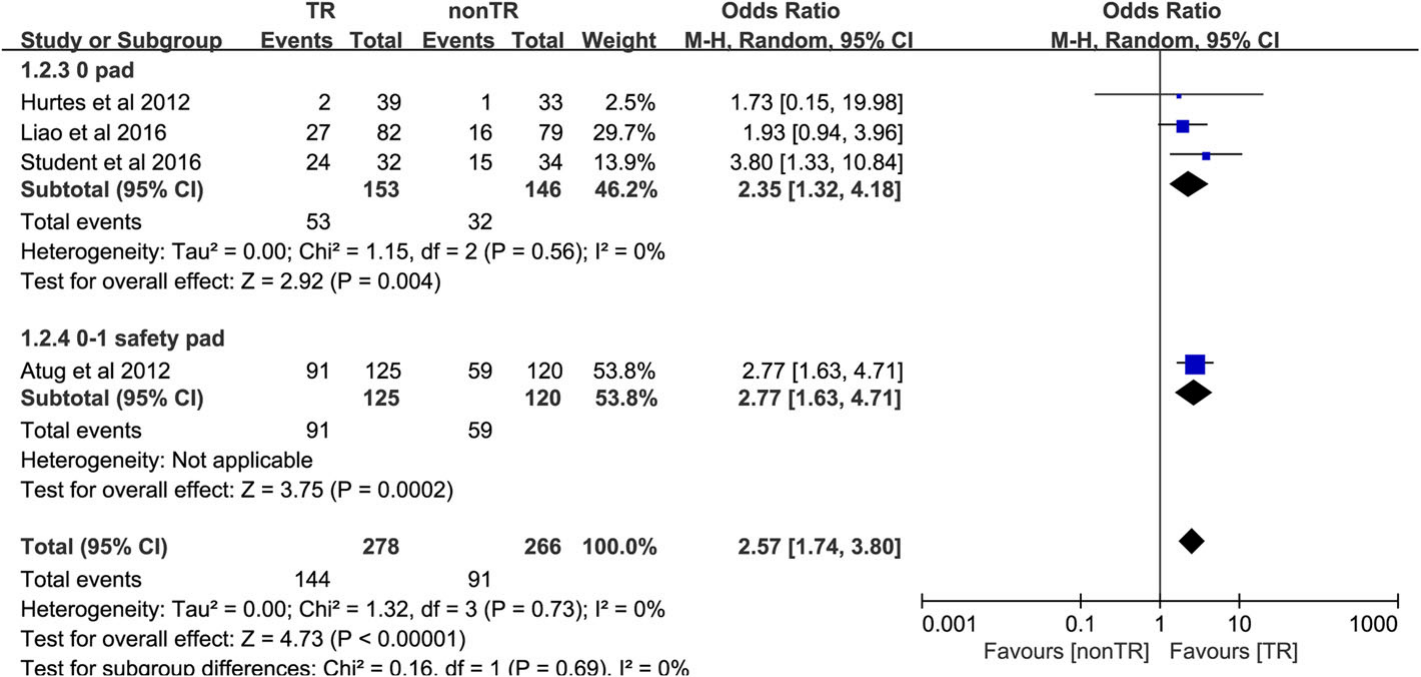
Forest plot of the odds of included studies comparing TR versus nonTR with respect to return to continence at 2 weeks. *CI* confidence interval, *OR* odds ratio, *RCT* randomized controlled trial, *TR* total pelvic floor reconstruction

Four trials [19, 20, 25, 27] reported the number of people returning to continence at 2 weeks. In the 0-pad subgroup, the cumulative results showed a statistically significant difference between the TR and nonTR groups in terms of return to continence at 2 weeks (OR 2.35, 95% CI 1.32 to 4.18; *P* = 0.004). In the 0–1 for safety pad subgroup, the cumulative results showed a statistically significant difference in favor of TR 2 weeks after RP (OR 2.77, 95% CI 1.63 to 4.71; *P* < 0.001). The overall cumulative results showed a statistically significant difference in favor of TR 2 weeks after RP (OR 2.57, 95% CI 1.74 to 3.80; *P* < 0.001) (Fig. 3).

#### Return to continence at 4 weeks

**Fig. 4:**
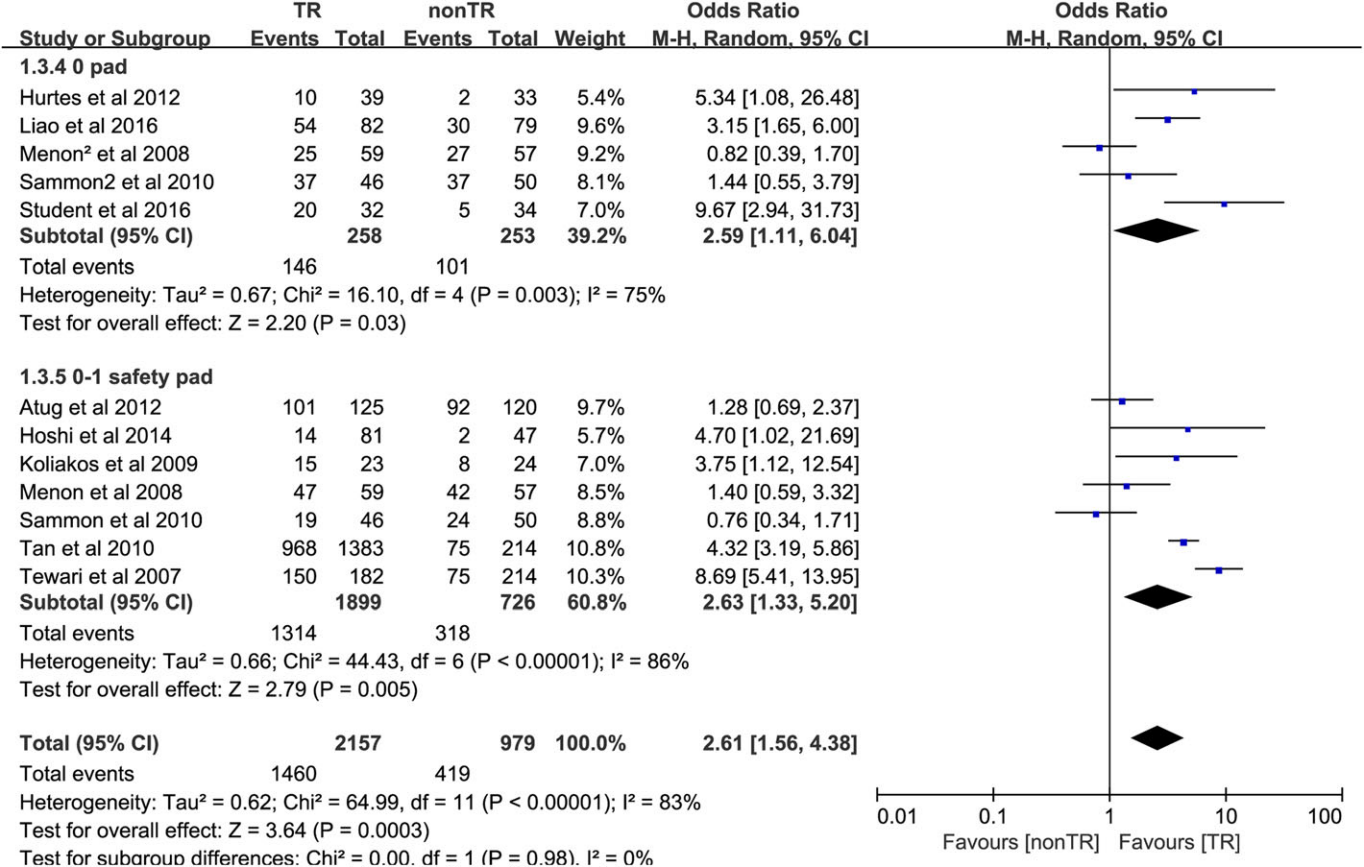
Forest plot of the odds of included studies comparing TR versus nonTR with respect to return to continence at 4 weeks. *CI* confidence interval, *OR* odds ratio, *RCT* randomized controlled trial, *TR* total pelvic floor reconstruction

Ten studies [10, 18–23, 25–27] comprising 12 trials reported the number of people returning to continence at 4 weeks. In the 0-pad subgroup, the cumulative results showed a statistically significant difference between the TR and nonTR groups in terms of return to continence at 4 weeks (OR 2.59, 95% CI 1.11 to 6.04; *P* = 0.03). In the 0–1 for safety pad subgroup, the cumulative results showed a statistically significant difference in favor of TR 4 weeks after RP (OR 2.63, 95% CI 1.33 to 5.20; *P* = 0.005). The overall cumulative results showed a statistically significant difference in favor of TR 4 weeks after RP (OR 2.61, 95% CI 1.56–4.38; *P* < 0.001) (Fig. 4).

#### Return to continence at 12 weeks

**Fig. 5:**
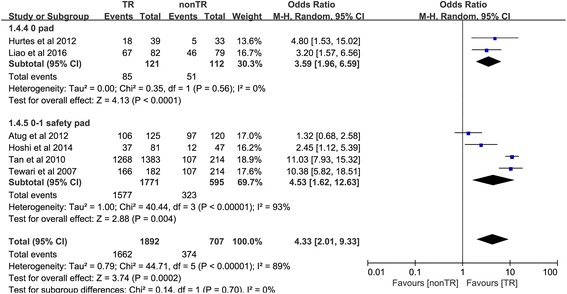
Forest plot of the odds of included studies comparing TR versus nonTR with respect to return to continence at 12 weeks. *CI* confidence interval, *OR* odds ratio, *RCT* randomized controlled trial, *TR* total pelvic floor reconstruction

Six trials [10, 18–20, 25, 26] reported the number of people returning to continence at 12 weeks. In the 0-pad subgroup, the cumulative results showed a statistically significant difference between the TR and nonTR groups in terms of return to continence at 12 weeks (OR 3.59, 95% CI 1.96 to 6.59; *P* < 0.001). In the 0–1 for safety pad subgroup, the cumulative results showed a statistically significant difference in favor of TR 12 weeks after RP (OR 4.53, 95% CI 1.62 to 12.63; *P* = 0.004). The overall cumulative results showed a statistically significant difference in favor of TR 12 weeks after RP (OR 4.33, 95% CI 2.01–9.33; *P* < 0.001) (Fig. 5).

#### Return to continence at 24 weeks

**Fig. 6:**
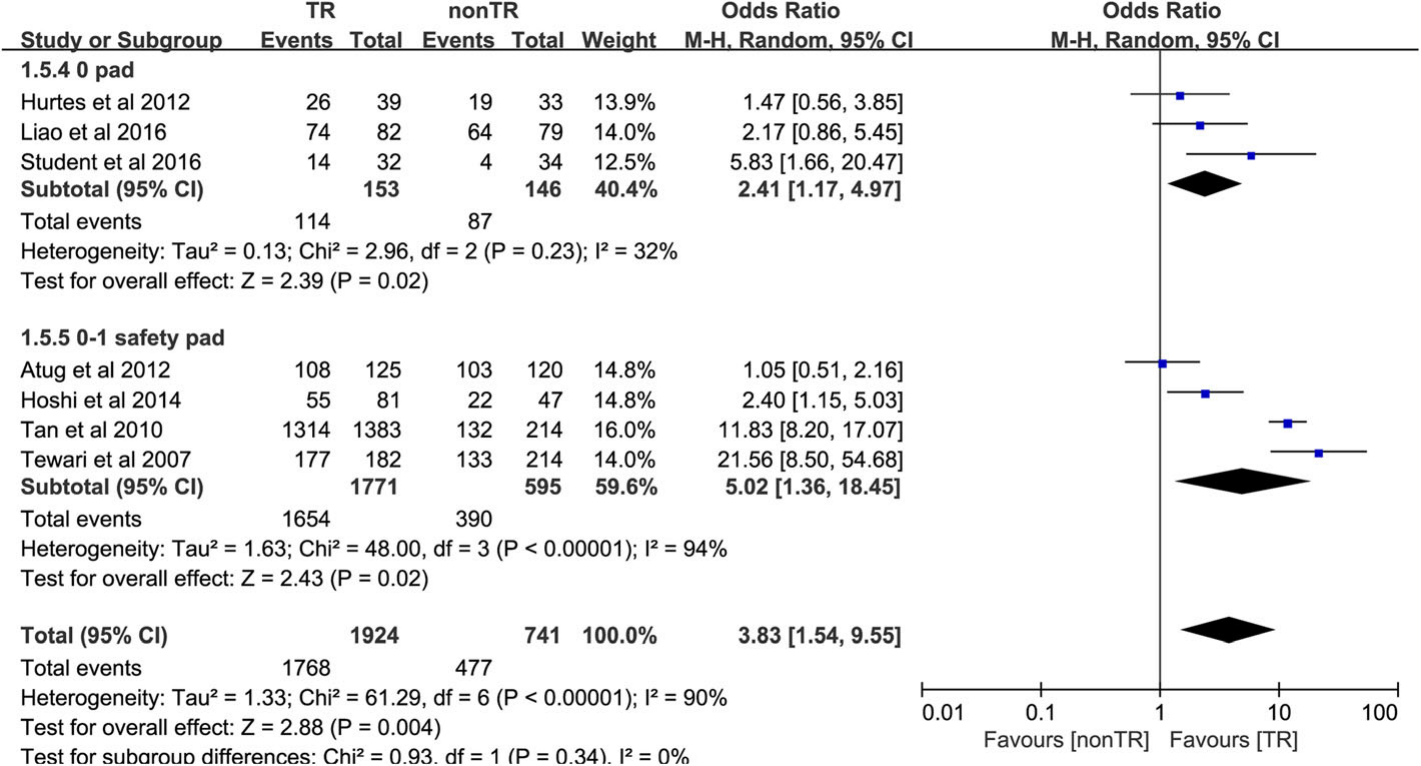
Forest plot of the odds of included studies comparing TR versus nonTR with respect to return to continence at 24 weeks. *CI* confidence interval, *O*R odds ratio, *RCT* randomized controlled trial, *T*R total pelvic floor reconstruction

Seven trials [10, 18–20, 25–27] reported the number of people returning to continence at 24 weeks. In the 0-pad subgroup, the cumulative results showed a statistically significant difference between the TR and nonTR groups in terms of return to continence at 24 weeks (OR 2.41, 95% CI 1.17 to 4.97; *P* = 0.02). In the 0–1 safety pad subgroup, the cumulative results showed a statistically significant difference in favor of TR 24 weeks after RP (OR 5.02, 95% CI 1.36 to 18.45; *P* = 0.02). The overall cumulative results showed a statistically significant difference in favor of TR 24 weeks after RP (OR 3.83, 95% CI 1.54–9.55; *P* = 0.004) (Fig. 6).

#### Return to continence at 52 weeks

**Fig. 7:**
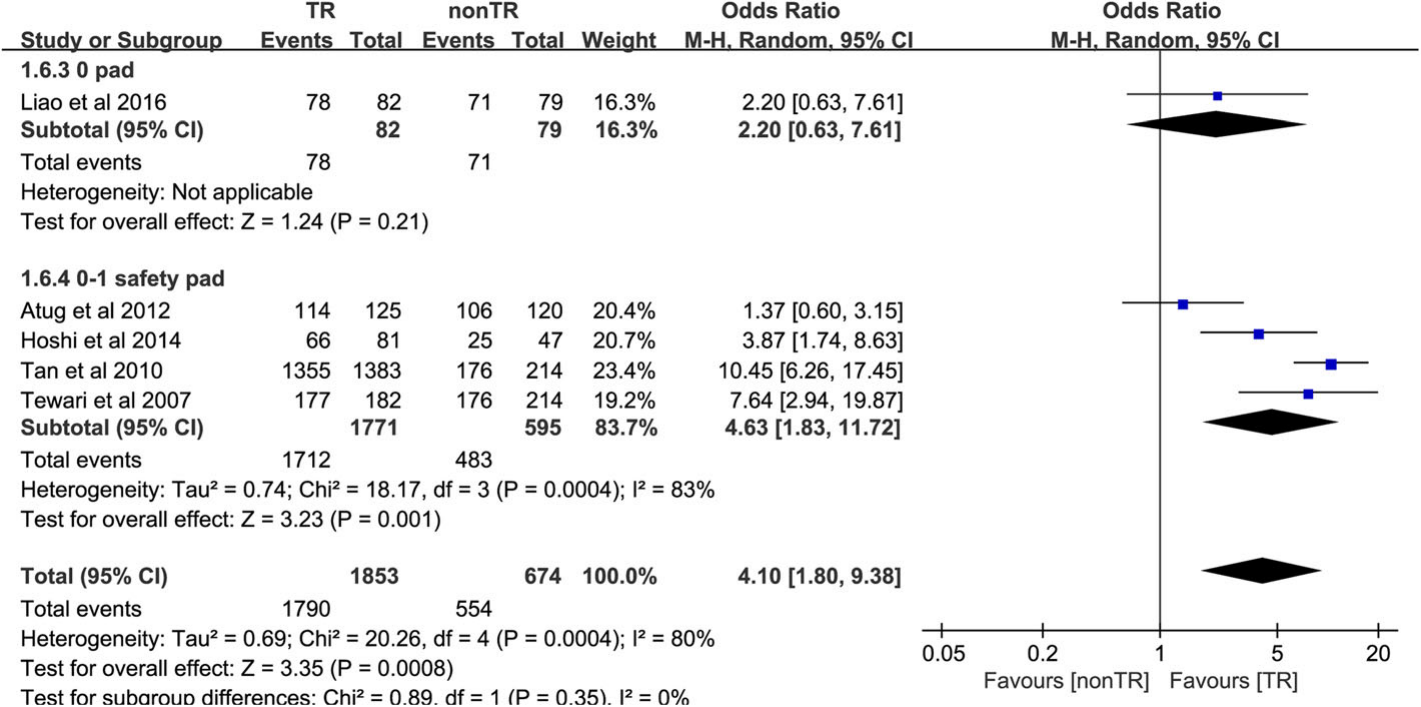
Forest plot of the odds of included studies comparing TR versus nonTR with respect to return to continence at 52 weeks. *CI* confidence interval, *OR* odds ratio, *RCT* randomized controlled trial, *TR* total pelvic floor reconstruction

Five studies [10, 18, 19, 25, 26] reported the number of people returning to continence at 52 weeks. In the 0-pad subgroup, the cumulative results showed no statistically significant difference between the TR and nonTR groups in terms of return to continence at 52 weeks (OR 2.20, 95% CI 0.63 to 7.61; *P* = 0.21). In the 0–1 safety pad subgroup, the cumulative results showed a statistically significant difference in favor of TR 52 weeks after RP (OR 4.63, 95% CI 1.83 to 11.72; *P* = 0.001). The overall cumulative results showed a statistically significant difference in favor of TR 52 weeks after RP (OR 4.10, 95% CI 1.80–9.38; *P* < 0.001) (Fig. 7).

### Positive surgical margin rate

**Fig. 8:**
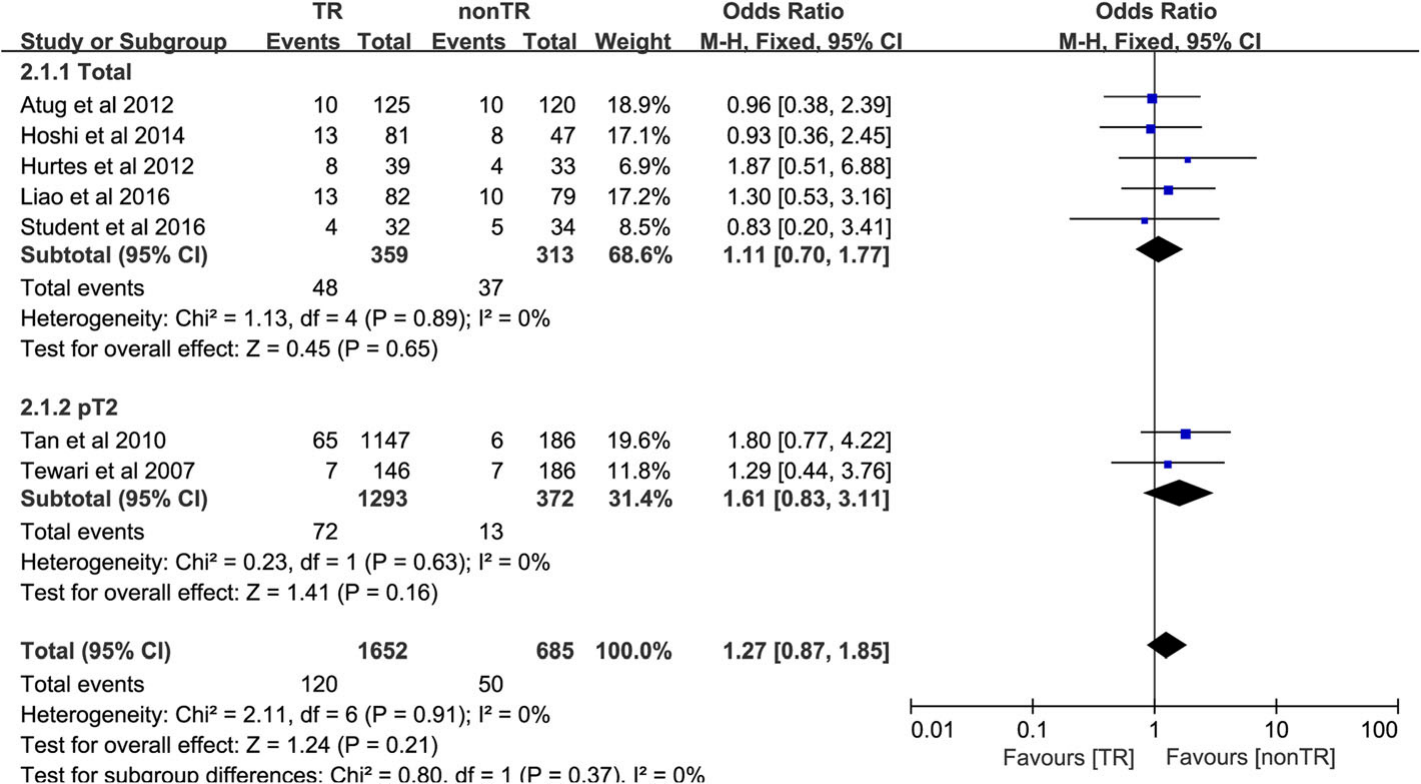
Forest plot of the odds of included studies comparing TR versus nonTR with respect to positive surgical margin rates. *CI* confidence interval, *OR* odds ratio, *RCT* randomized controlled trial, *TR* total pelvic floor reconstruction

The positive surgical margin (PSM) rates of all enrolled trials are summarized in Table 4. In the total pathological stage subgroup, the pooled results demonstrated that there was no significant difference between the two groups (OR 1.11, 95% CI 0.70 to 1.77, *P* = 0.65). In the pT2 subgroup, the pooled results demonstrated that there was also no significant difference between the two groups (OR 1.61, 95% CI 0.83 to 3.11, *P* = 0.16). The pooled results of both subgroups indicated that there was no significant difference between the two groups (OR 1.27, 96% CI 0.87 to 1.85; *P* = 0.21) (Fig. 8).

### Operation time, estimated blood loss, duration of catheterization, and complication rate

**Fig. 9:**
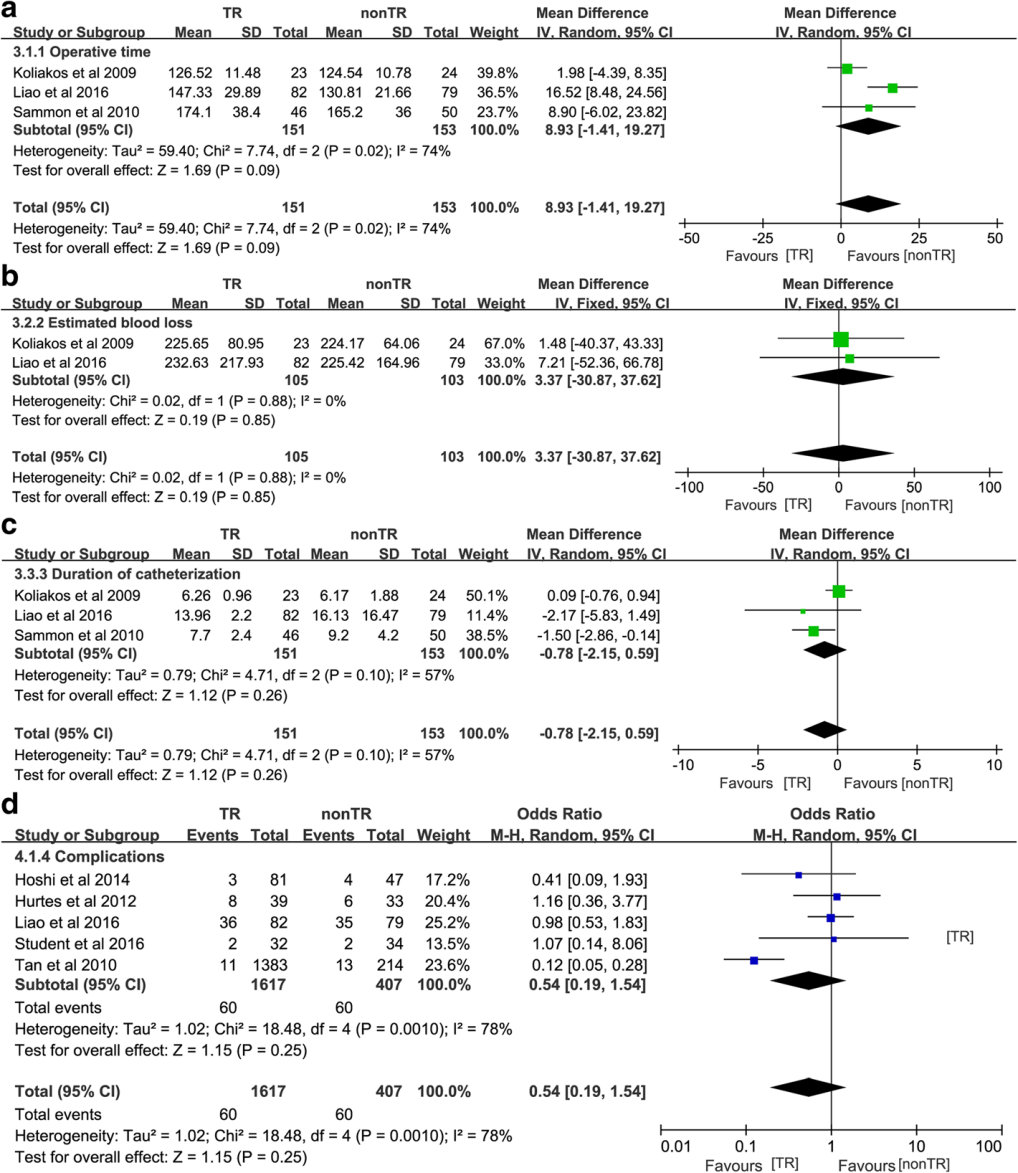
Forest plots of the odds or mean difference of included studies comparing TR versus nonTR with respect to **a** operative time, **b** estimated blood loss, **c** duration of catheter, and **d** complications. *CI* confidence interval, *SD* standard deviation, *OR* odds ratio, *TR* total pelvic floor reconstruction

The perioperative outcome measures and complications are summarized in Table 4. The pooled results demonstrated that there was no significant difference between the two groups in terms of operative time (OR 8.93, 95% CI − 1.41 to 19.27, *P* = 0.09) (Fig. 9a), estimated blood loss (OR 3.37, 95% CI − 30.87 to 37.62, *P* = 0.85) (Fig. 9b), and duration of catheterization (OR − 0.78, 95% CI − 2.15 to 0.59, *P* = 0.26) (Fig. 9c). The complications of the seven trials are summarized in Table 4. The pooled results demonstrated that there was no significant difference between the two groups in terms of complications (OR 0.54, 95% CI 0.19 to 1.54; *P* = 0.25) (Fig. 9d). The cumulative results demonstrated that the TR technique is a simple method associated with early recovery from UI with no increase in operation time, estimated blood loss, duration of catheterization, and complication rate.

## Discussion

UI plays an important role in reducing the QoL and raising the cost of care after RP [18]. Several technical modifications have been used to improve postoperative incontinence. However, variability in the rate of UI following RP remains one of the most important and significant functional complications.

In our meta-analysis, TR appeared to improve not only short-term urinary continence but also long-term urinary continence. To the best of our knowledge, the usefulness of the TR technique for improving long-term urinary continence remains controversial. Our meta-analysis demonstrated statistically significant differences in favor of the TR technique for urinary continence recovery. No statistically significant differences between the TR and nonTR groups were also observed for PSM rate, operation time, estimated blood loss, duration of catheterization, or complication rate.

The prevalence of UI after RP is significantly affected by participant preoperative demographics, surgeon experience, surgical technique, definition of continence, data collection tools, and differences in follow-up intervals [28] (Tables 3, 4, 5, and 6). Specifically, the continence rates varied, and there were discrepancies in the definition of continence. Menon et al. [22] found that 7-day urinary continence rates were 20 versus 16% in cases using a no-pad definition and 54 versus 51% in cases using a 0–1 for safety definition for patients underwent TR and nonTR, respectively. The 31-day urinary continence rates were 42 versus 80% in cases using a no-pad definition and 47 versus 74% in cases using a 0–1 pad for patients underwent TR and nonTR, respectively. However, a multicenter study is needed to externally validate these data.

There were several limitations in this study. First, the technique of TR was not standardized in all studies. The differences in surgical techniques for each surgical reconstruction process were reported accordingly. Student et al. [27] sutured the arcus tendineus to the bladder neck served as the anterior fixation and formed the dorsal support for the urethrovesical anastomosis using the fibers of the median dorsal raphe, retrotrigonal layer, Denonvillier’s fascia, and the levator ani muscle. Liao et al. [19] began the total reconstruction with posterior reconstruction that was accomplished by suturing the bladder musculature, Denonvillier’s fascia, and musculofascial plate posterior to the urethra. Then the arcus tendineus and puboprostatic plate were reattached to the bladder neck. Second, the definition of urinary continence recovery was not uniform in every study. Different methods were used to evaluate urinary continence recovery including the number of pads; pad tests; pad weight; the International Consortium on Incontinence Questionnaire, short form (ICIQ-SF); International Prostate System Score (IPSS); International Index of Erectile Function (IIEF-5) questionnaire; and European prospective investigation into cancer and nutrition (EPIC) questionnaire. Third, the different study designs could have influenced the outcomes of this study. A nerve-sparing technique was used in the surgical procedure by Liao et al. [19] and Atug et al. [25], while a different nerve-sparing technique was used by Menon et al. [22] and Sammon et al. [23]. Because of the small number of reported trials, we did not distinguish RCTs from retrospective studies.

## Conclusions

TR appeared to be associated with an advantage for urinary continence recovery 1, 2, 4, 12, 24, and 52 weeks after RP. For the first time, our meta-analysis demonstrated a significant advantage in favor of TR in terms of both short- and long-term (1, 2, 4, 12, 24, and 52 weeks) urinary continence recovery. However, this result needs to be validated in further multicenter, prospective, randomized, controlled studies. Methodological factors need to be taken into account when interpreting the cumulative results.
